# Supporting Pre-Exposure Prophylaxis Uptake: Exploring Social Network Characteristics among Black Women and Acceptability of Social Network Strategies

**DOI:** 10.3390/healthcare12171769

**Published:** 2024-09-04

**Authors:** Amy K. Johnson, Samantha A. Devlin, Miranda Hill, Emily Ott, Eleanor E. Friedman, Sadia Haider

**Affiliations:** 1 The Potocsnak Family Division of Adolescent and Young Adult Medicine, Ann & Robert H. Lurie Children’s Hospital, Chicago, IL 60611, USA; 2 Feinberg School of Medicine, Northwestern University, Chicago, IL 60611, USA; 3 Section of Infectious Diseases and Global Health, Department of Medicine, University of Chicago, Chicago, IL 60637, USA; 4 School of Medicine, University of California, San Francisco, Oakland, CA 94612, USA; 5 Department of OB/GYN, Rush University Medical Center, Chicago, IL 60612, USA

**Keywords:** black/African American, women, HIV prevention, pre-exposure prophylaxis (PrEP), social networks

## Abstract

Black women continue to be disproportionally burdened by HIV. Pre-exposure prophylaxis (PrEP) is an effective HIV prevention option, which is underused by Black women. While social network interventions (SNIs) have been widely researched and implemented among some groups vulnerable to HIV, little is known about social network characteristics among Black women. To learn more about the social networks of Black women vulnerable to HIV and their knowledge of and interest in PrEP, we conducted a cross-sectional survey among 109 Black women aged 18–45 years attending a family planning clinic in Chicago, Illinois. In our study, 44% of women reported that they were moderately to extremely concerned about HIV. Over half of participants (53%) had a small personal network size (i.e., less than two). No statistically significant associations between having larger network sizes and having previously heard of PrEP, having an interest in starting PrEP, or having good PrEP knowledge were detected. Open-ended responses revealed high levels of trust in network connections with matters related to sexual health. Additionally, nearly all (94%) of women reported that SNIs were a good idea to promote PrEP. Future network studies are needed to inform the development of effective intervention strategies for women.

## 1. Introduction

HIV (human immunodeficiency virus) is a virus that attacks the body’s immune system. If untreated, it can lead to AIDS (acquired immunodeficiency syndrome) [1]. In the United States, women accounted for 18% of all new HIV diagnoses in 2021 [2,3]. Black women continue to face a severe burden of HIV compared to other racial and ethnic groups. For example, Black women accounted for over half (54%) of all new HIV infections among women in 2021 [2,3]. To address this disparity, it is important to develop and test effective strategies for promoting HIV prevention methods, particularly the use of an HIV prevention medication called pre-exposure prophylaxis (PrEP).

Previous research has shown that PrEP knowledge among women is low, but women consistently report high interest in using PrEP and positive attitudes toward PrEP [4,5]. Positive perceptions of PrEP among women suggest that awareness, rather than willingness, may be a key barrier to PrEP uptake, and that dissemination efforts to educate women on PrEP have been insufficient [4,6,7]. A potential strategy to disseminate PrEP information to women is the use of social networks, whose dynamics can strongly influence a person’s vulnerability to HIV and willingness to engage in HIV prevention [8].

Social network interventions (SNIs) are built on a network theory that asserts that an individual’s beliefs and behaviors can be shaped by the social norms, behaviors, and reward systems of their social network members [9]. Studies have demonstrated that the higher the prevalence of social network members who adopt an innovation, the more likely a network member will be to adopt (and know about) an innovation [10]. Indeed, elements of social network structure, including network size (number of network members) and quality of relationship (measured by trust), have been shown to influence HIV risk and prevention behaviors [11]. As such, they have been effective among men who have sex with men (MSM), transgender women, homeless youth, and people who inject drugs [10,12].

Despite over two decades of research on social network characteristics and interventions, few studies have explored the social networks of women in relation to understanding their vulnerability to HIV and increasing awareness of PrEP [13,14,15,16]. Felsher et al. found that women’s decision to discuss PrEP within their social network was largely explained by social network-level factors [15]. For instance, participants provided PrEP information to their female peers when (1) they perceived their peers as vulnerable to HIV or (2) they were emotionally close to peers and disclosed their PrEP use to peers to increase social connectedness and support. A few PrEP conversations resulted from unintentional PrEP pill discovery or preemptively to avoid the negative outcomes of unexpected discovery (i.e., judgment, loss of relationship, or financial repercussions). There is a clear need to develop specific recommendations about which women would be most successful in engaging network members and how women should engage with their networks around PrEP (i.e., women who are interested in PrEP may be embedded within social and normative contexts that can encourage or impede PrEP uptake) [15,16].

Due to their increased vulnerability to HIV, it is important to determine if the social networks of Black women specifically can be leveraged to support HIV prevention and PrEP uptake. Neblett et al. assessed social network characteristics and HIV risk behaviors among 513 African American women in Baltimore, Maryland [14]. The impact of social network variables, including total network size, emotional support, financial support from network members, drug use among network members, and conflict with members of their network, was assessed in relation to HIV risk behaviors. Having an at-risk sexual partner (i.e., someone who injected/used drugs, was living with HIV, or had been recently diagnosed with a sexually transmitted infection) in the past 90 days was associated with having a larger social network and having more network members who used heroin or cocaine. Taken together, previous findings on SNIs among women demonstrate the need for increased efforts to promote information sharing and research to determine the key factors influencing HIV prevention choices among women [11,17,18].

Given the potential for SNIs to promote PrEP uptake among Black women, and the scarcity of research in this area, this study had two primary aims: (1) to explore and describe social network characteristics (e.g., types and numbers of people in their trusted networks) among Black women attending a family planning clinic and (2) to assess the perception of SNIs among participants, with the ultimate objective of determining if SNIs are feasible and acceptable strategies to address HIV vulnerability and increase PrEP awareness among Black women.

## 2. Materials and Methods

### 2.1. Data Collection

A cross-sectional survey was conducted with women attending the University of Chicago Ryan Center family planning clinic. Potential participants were approached by study staff in clinic waiting rooms. Recruitment flyers were also posted in the clinic, and potential participants could call or email the research team to learn more about this study. Interested women were screened for survey participation and gave consent if eligible. Eligibility was determined as follows: speaks English, self-identifies as Black, is between the ages of 18 and 45 years (inclusive), lives in Chicago, and can report sexual activity within the previous 12 months. This study was completed in a private room/area of the clinic waiting room and consisted of a quantitative survey and a series of semi-structured open-ended questions. Following survey completion, participants received a USD 20 gift card and local PrEP-related resources. The survey data were collected and managed using REDCap electronic data capture tools hosted at University of Chicago. All participants completed written informed consent prior to engaging in this study; the Institutional Review Boards from the University of Chicago (IRB17-0984) and Lurie Children’s Hospital (IRB17-1410) reviewed and approved this study.

All data were self-reported. The survey was designed to capture information about PrEP knowledge, acceptability, barriers and facilitators to uptake, demographic and behavioral domains, and information about trusted networks. Demographic characteristics included age and neighborhood. We assessed PrEP knowledge with a score based on a series of six true/false questions (e.g., “PrEP can be taken by both men and women to prevent HIV infection”). A higher score indicated a greater level of PrEP knowledge. We measured the size and characteristics of three types of trusted networks that Black women regularly consulted, including personal, sexual health, and peer networks (Black women), as well as how many of those people were related or connected to one another. Open-ended questions measured participants’ acceptability of SNIs and included items on the quality of social network ties, accessing advice and sexual health information, and feedback on the intervention strategy. Responses to open-ended items were audio-recorded and then transcribed verbatim by a study team member.

### 2.2. Data Analysis

Descriptive statistics included frequencies and percentages for categorical variables, or medians and interquartile ranges (IQRs) for continuous variables. Bivariate analyses were used to detect differences between outcome variables and network sizes. We dichotomized the total number of network contacts into two categories: below/at the median or above the median. We examined associations with the following variables: having heard of PrEP prior to this study, willingness to start PrEP in the next three months, low or high PrEP knowledge, and the total count of barriers to PrEP uptake. Associations were modeled using logistic regression and odds ratios and 95% confidence intervals (ORs, 95%CIs) or by linear regression with Beta coefficients and standard errors. Quantitative analysis was conducted in SAS version 9.4 (SAS Institute, Cary, NC, USA). Open-ended data transcripts were managed and analyzed using Dedoose software version 9.0.90 (SocioCultural Research Consultants, LLC, Los Angeles, CA, USA). Guided by a conventional content analysis framework, the study team reviewed the open-ended responses and developed a coding scheme. The responses were then coded by two team members. The results of the coding differed in less than 5% of the data. In cases where coding differed, the study team reviewed the data and assigned a code by consensus [19].

## 3. Results

In total, 109 Black women completed the study from February to July 2018. The median age was 28 years (IQR 25–33). The participants lived in areas with diverse HIV prevalence [20], with just under a third (28%) living in the lower HIV prevalence neighborhoods in the city of Chicago (26.2–669.6 cases per 100,000 residents), 56% living in higher HIV prevalence neighborhoods in Chicago (666.7–2004.9 per 100,000 residents), and 16% living outside of Chicago in a range of prevalence areas (Table 1).

Only 11% of participants thought they had a 50% or higher risk of getting HIV, yet a significant proportion of participants (44%) were moderately/extremely concerned about getting HIV and were concerned about the HIV burden within their community (67%). Moreover, participants who lived in an area of Chicago with a higher prevalence of HIV were significantly more likely to think that HIV was a problem in their neighborhoods than those that lived in areas with a lower prevalence of HIV in Chicago or outside of the city (Kendall’s tau two-sided probability = 0.02).

Prior to this study, only 37% of participants had heard of PrEP. After PrEP was introduced to participants, 32% expressed interest in starting PrEP in the next three months (very likely or somewhat likely to start PrEP). Barriers to PrEP uptake were common, with a median of 2 (IQR 1,3). Commonly reported barriers included side effects (51%), the newness of the drug (45%), and cost (33%). Knowledge of PrEP was generally high, with a median score of 5 (IQR 4, 5) and 74% of participants with good PrEP knowledge.

Participants reported a wide range of people within their network (Table 2). In general, people had a low median network size and tight interquartile intervals regardless of the particular network measured (personal, sexual health, and peer/Black women). Personal network size had a median of 2 (IQR 2–3); sexual health network size had a median of 2 (IQR 1–2); and Black female network size had a median of 3 (IQR 1–5). When dichotomized to small or large network sizes, the majority of participants had small networks for each type of network: 53% had a small personal network size; 79% of participants had a small sexual health network size; and 58% had a small network size of Black women (Table 3). When asked to choose the most trusted member of each participants’ combined network, most respondents chose their mother (74%), a sister (56%), or a friend (46%).

Overall, no associations between having large network sizes (personal or private network size, sexual health network size, and Black female network size) and having previously heard of PrEP, interest in starting PrEP, or good PrEP knowledge were seen (Table 4). Additionally, personal or private network size, sexual health network size, and Black female network size were also not associated with a total count of barriers to PrEP uptake (Table 5).

### Open-Ended Responses

When asked whether or not participants trusted their network connections with matters related to sexual health, 87% of the sample reported that they did trust their network connection: “*Well, I say yes because I am very open about sexual health. I feel like that’s important...I’ve been knowing them all my life…we talk about everything*”. The remaining 13% of participants responded saying that they “sometimes” trusted connections or that they did not trust connections in regard to sexual health matters: “*that’s* [sexual health] *like too personal, so I wouldn’t discuss with a cousin, an aunt, or anything like that*…”.

The participants were asked how they would like to receive information about PrEP. Thirty-four percent of participants reported they wanted to receive PrEP information from their doctors, stating they trusted their doctor and would like medical-related advice to come from them. Twenty-nine percent of participants reported being comfortable with multiple sources and highlighted the fact that the more sources that had PrEP information, the more likely they would be to access and understand the information: “*All the above—the more information I know, the more I can protect myself* [from HIV]”. Twenty-three percent of participants reported they wanted to hear about PrEP from community members via face-to-face interactions: “*Me, personally, I like hearing it from people who actually have been through it. ‘Cause I feel like sometimes the doctors sugar coat stuff so you won’t be as paranoid, so I prefer to hear from someone who already been through it or took it* [PrEP]”.

The majority (94%) of participants reported they felt SNIs were a good idea to use with Black women to promote PrEP. The responses highlighted trust, confidentiality, and the strength of existing relationships, as one participant stated, “*If it’s* [PrEP information] *coming from someone you trust, it’s more believable, so the mistrust is not really there.* [There is] *already a connection there*”. Participants who thought SNIs would not work discussed an overall lack of belief, discomfort with talking about sexual health, and the idea that HIV information may need to come from a doctor or other medical provider: “*A lot of people are stubborn, if it’s not brought up to me by a doctor, then I don’t want to hear it*”. Sixty-five percent of participants agreed they would be interested in being trained to share PrEP information with their networks. Those who did not agree gave feedback that they were not the type to discuss these matters or that they were uncomfortable with the commitment of being an influencer (i.e., someone who discusses sexual health matters/HIV prevention and promotes PrEP with their peers) within their network.

## 4. Discussion

Participants in our study reported networks that were limited in size and density but ranked high on quality (trust) and duration (consisting mainly of family members). These findings are consistent with egocentric network analyses of motivations for PrEP-related communication among women vulnerable to HIV, with participants on average naming three network members [15] and women reporting their preference to discuss PrEP with close, supportive, and trusted network members [16]. Additionally, stronger emotional closeness has been associated with a higher willingness to share PrEP-related information among networks of women [21]. Despite reporting small network sizes, once hearing about SNIs, the majority of participants agreed that it would be a good intervention strategy to promote knowledge of PrEP among Black women.

The participants’ perception of their neighborhood’s risk of HIV aligns with their reported living areas (e.g., participant’s neighborhood perceived as vulnerable to HIV, participant lived in high-prevalence neighborhood), indicating an awareness of trends in HIV. However, despite this awareness and the reality of living in a high-prevalence community, only two participants reported having any network members living with HIV. Participants may not have felt comfortable sharing the HIV status of their close network connections or may have network members who have not disclosed their status. Increasing HIV risk perception in vulnerable communities can be an effective starting point for health promotion and PrEP awareness interventions [22].

Nearly a quarter of women wanted to hear about PrEP from trusted community sources via face-to-face interactions, and the overwhelming majority of participants thought SNIs would be an effective way to promote PrEP among Black women, particularly if they utilized existing relationships. Moreover, the majority of women said they would be interested in being trained to share PrEP information with their networks. Our findings contribute to the existing literature regarding Black women’s perceptions of social network-based PrEP interventions. For example, previous studies have investigated the acceptability and feasibility of a social network-based PrEP intervention in a beauty salon using hair stylists to improve PrEP uptake among Black women. These studies have demonstrated that social networks are pre-formed, developed, and nurtured in a salon and that social networks positively influence information-sharing, specifically with respect to health-related topics [23,24]. The participants in this study also reported wanting to hear about PrEP from their doctors and from many different sources. Therefore, coupling SNI strategies that utilize community sources (i.e., a salon-based intervention) with provider training and public health detailing could be an effective approach to HIV prevention among women [25].

As our study collected descriptive network data, future research should collect complete social network data to provide more information on the exact dimensions of the social networks of Black women, including the number of network members, and whether or not networks of different Black women connect. Intervention strategies could leverage smaller networks by focusing on tailored messages and using trusted shared network members, such as community doctors, pastors, and hair stylists. An intervention that uses trusted community-based networks rather than personal networks may help to facilitate discussions of sensitive topics such as HIV prevention and PrEP. Additional SNI strategies such as increasing the number of network influencers or leveraging mobile health (i.e., mHealth) devices to support influencer messaging could be employed if the networks of Black women prove less interconnected.

Unlike other studies, we did not identify significant associations between network size and PrEP knowledge or intent to take PrEP among our participants [9]. This may be due to our sample reporting small network sizes. The study results need to be interpreted with the following limitations in mind. First, our sample size was small and homogenous, which limited its statistical power and the ability to detect subgroup differences or build a multivariable model. Second, our sample was recruited from a single clinic in a large Midwestern city and thus should not be interpreted to be generalizable to all family planning patients, as regional variations may exist. It should be noted that all participants were recruited in a clinic setting and therefore responses could vary from a community-based sample. Women who are not engaged in healthcare may be more likely to prefer their social networks as a source for PrEP information compared to women who are engaged in healthcare. Finally, all data were self-reported and therefore may be subject to social desirability. To mitigate socially desirable responses, the data were collected via computer-assisted self-interviewing methods. Despite the above limitations, our findings make important contributions to the literature given the use of multimethod, quantitative, and qualitative approaches, which have scarcely, if at all, been used to draw critical insights on the intersection between HIV risk, PrEP awareness, social networks, and network-based intervention preferences among Black women. Altogether, these findings among Black women who live in high HIV prevalence settings emphasize the importance of continued investigation into tailored network-based PrEP interventions for this key group.

## 5. Conclusions

Culturally relevant interventions are needed to support Black women across the PrEP care continuum. This sample of Black women reported small network sizes but expressed high levels of trust with the people within their networks. There was a high acceptability of using SNI strategies for promoting PrEP among Black women and a high interest in being trained to share PrEP information with their networks. Network studies can inform the adaptation of existing evidence-based HIV prevention intervention strategies for Black women and are a promising method for reducing the burden of risk among this high-priority population.

## Figures and Tables

**Table 1:** Demographic and behavioral factors among survey respondents (N = 109).

Demographic and Behavioral Factors	Median (IQR), Range or N (%)
Age (N = 108)	
Median (IQR), Range	28 (25, 33), 18–64
Neighborhood prevalence of HIV per 100,000	
26.2–359.1	6 (5.5%)
359.2–699.6	25 (22.9%)
699.7–1185.6	43 (39.5%)
1185.7–2004.9	18 (16.5%)
Outside of Chicago	17 (15.6%)
I think my chances of getting HIV are:	
Missing	2 (1.8%)
0%—There is no chance I will get HIV	72 (66.1%)
25%	23 (21.1%)
50%	11 (10.1%)
75%	1 (0.9%)
100%—I will definitely get HIV	0 (0.0%)
Getting HIV is something I am:	
Not concerned about	37 (33.9%)
A little concerned about	24 (22.0%)
Moderately concerned about	21 (19.3%)
Concerned about a lot	7 (6.4%)
Extremely concerned about	20 (18.4%)
I am concerned about high rates of HIV in my community:	
Disagree	5 (4.6%)
Undecided	31 (28.4%)
Agree	73 (67.0%)
Prior to this study, have you heard of PrEP (pre-exposure prophylaxis) or the use of medication to prevent HIV infection?	
Yes	40 (36.7%)
No	69 (63.3%)
How likely are you to start taking PrEP in the next 3 months?	
Unsure, somewhat unlikely, very unlikely	74 (67.9%)
Very likely, somewhat likely	35 (32.1%)
Most trusted people (free text answer)	
Mother	81 (74.3%)
Sister	61 (56.0%)
Friend	50 (45.9%)
Father	35 (32.1%)
Brother	34 (31.2%)
Cousin	33 (30.3%)
Grandmother	30 (27.5%)
Significant other/father of children	30 (27.5%)
Aunt	23 (21.1%)
Uncle	9 (8.3%)
Faith leader	8 (7.3%)
Co-workers	5 (4.69%)
Grandfather	3 (2.8%)
Which of the following are barriers for you to PrEP uptake? (Choose all that apply)	
Lack of communication among community members	25 (22.9%)
Mistrust of the medical community	16 (14.7%)
Cost	36 (33.0%)
Side effects	55 (50.5%)
Stigma	10 (9.2%)
Drug is too new	49 (45.0%)
Lack of housing	0 (0.0%)
Other	20 (18.4%)
PrEP can be acquired over the counter at any drug store without a prescription	
False	84 (77.1%)
True	22 (20.2%)
Missing	3 (2.7%)
PrEP needs to be taken daily in order to be effective	
False	8 (7.3%)
True	98 (89.9%)
Missing	3 (2.8%)
PrEP can be taken by both men and women to prevent HIV infection	
False	7 (6.4%)
True	99 (90.8%)
Missing	2 (2.8%)
PrEP is effective in stopping HIV transmission among people living with HIV	
False	66 (60.6%)
True	40 (36.7%)
Missing	3 (2.7%)
PrEP is only available for prevention HIV among gay and bisexual men	
False	102 (93.6%)
True	4 (3.7%)
Missing	3 (2.7%)
PrEP can be used to prevent infection after being exposed to HIV	
False	60 (55.1%)
True	46 (42.2%)
Missing	3 (2.7%)

**Table 2:** Participant responses to questions regarding the size and contents of their social networks (N = 109).

Questions Regarding Network Members	Median (IQR), Range
In the past 6 months, with how many people have you talked to about things that are personal or private? (N = 108)	2 (2, 3) 0–20
How many of these people know each other? (N = 94)	2 (2, 3) 0–20
How many of these people are family members? (N = 106)	1 (0, 2) 0–10
How many of these people are female? (N = 106)	2 (1, 3) 0–10
How many of these people are HIV-positive? (N = 105)	0 (0, 0) 0–2
In the past 6 months, with how many people have you talked to about matters related to your sexual health or sexual behavior? (N = 109)	2 (1, 2) 0–50
How many of these people know each other? (N = 59)	2 (2, 2) 0–10
How many of these people are family members? (N = 89)	1 (0, 1) 0–10
How many of these people are female? (N = 89)	2 (1, 2) 0–38
How many of these people are HIV-positive? (N = 88)	0 (0, 0) 0–0
How many Black women do you know who are about your age and live in the same area you do, with whom you speak to on a regular basis? (N = 109)	3 (1, 5) 0–1500
Number of total barriers reported (N = 109)	2 (1, 3), 0–8
Knowledge score (N = 106)	5 (4, 5), 0–6

**Table 3:** Number of participants who reported small or large networks based on the median number of network members for various network types (N = 109).

Network Type	N (%)
Personal or private network size	
≤2	58 (53.2%)
>2	51 (46.8%)
Sexual health network size	
≤2	86 (78.9%)
>2	23 (21.1%)
Black women network size	
≤3	63 (57.8%)
>3	46 (42.2%)

**Table 4:** Logistic regression models for network size (dichotomized at the median number of network members) and having previously heard of PrEP, willingness to start PrEP, or good PrEP knowledge (N = 109).

Logistic Regression Odds Ratios	Heard PrEP OR (95%CI), Wald Chi Squared *p*-Value	Start PrEP OR (95%CI), Wald Chi Squared *p*-Value	High PrEP Knowledge OR (95%CI), Wald Chi Squared *p*-Value
Personal or private network size			
≤2	Reference	Reference	Reference
>2	0.8 (0.4, 1.7), 0.5	0.5 (0.2, 1.1), 0.1	1.2(0.5, 2.8), 0.8
Sexual health network size			
≤2	Reference	Reference	Reference
>2	1.8 (0.7, 4.6), 0.2	1.88 (0.73, 4.84), 0.19	0.6 (0.2, 2.0), 0.4
Black women network size			
≤3	Reference	Reference	Reference
>3	1.2 (0.6, 2.6), 0.7	1.0 (0.5, 2.4), 0.9	0.9 (0.4, 2.2), 0.8

**Table 5:** Linear regression models between various network sizes and the total number of barriers to PrEP uptake.

Type of Network	Beta Coefficient (Standard Error), *p*-Value
Personal or private network size	
≤2 vs. >2	−0.1 (0.2), 0.6
Sexual health network size	
≤2 vs. >2	−0.3 (0.3), 0.4
Black women network size	
≤3 vs. >3	0.4 (0.2), 0.1

## Data Availability

The raw data supporting the conclusions of this article can be made available by the authors upon reasonable request.
